# Gonorrhœa and Syphilis

**Published:** 1878-04

**Authors:** 


*A Treatise on Gonorrhœa and Syphilis.* By SILAS DURKEE, M. D. Sixth Edition. Philadelphia: Lindsay & Blakiston. 1877. Buffalo: T. H. Butler. pp. 467. Price, $3.50.

This work has become a standard authority and needs no recommendation to keep it before the profession. It has been tried and has not failed. It is the work of a master who after spending thirty years in study upon these important subjects puts forth a volume as the embodiment of his matured opinion. The desire of the author was to furnish a book that would be permanently useful, we feel sure that his object has been fulfilled. C. A. W.

